# Cognitive lifestyle jointly predicts longitudinal cognitive decline and mortality risk

**DOI:** 10.1007/s10654-014-9881-8

**Published:** 2014-02-28

**Authors:** Riccardo E. Marioni, Cecile Proust-Lima, Helene Amieva, Carol Brayne, Fiona E. Matthews, Jean-Francois Dartigues, Helene Jacqmin-Gadda

**Affiliations:** 1 Department of Public Health and Primary Care, University of Cambridge, Cambridge, CB2 0SR UK; 2 INSERM, Centre INSERM U897, 33000 Bordeaux, France; 3 University of Bordeaux, ISPED, Centre INSERM U897, 33000 Bordeaux, France; 4 Service de Neurologie, Department of Clinical Neurosciences, CHU, Pellegrin, 33076 Bordeaux, France; 5 MRC Biostatistics Unit, Institute of Public Health, Cambridge, CB2 0SR UK; 6 Department of Psychology, Centre for Cognitive Ageing and Cognitive Epidemiology, University of Edinburgh, 7 George Square, Edinburgh, EH8 9JZ UK

**Keywords:** Cognitive lifestyle, Mortality, Cognitive decline, Longitudinal modelling

## Abstract

Cognitive lifestyle measures such as education, occupation, and social engagement are commonly associated with late-life cognitive ability although their associations with cognitive decline tend to be mixed. However, longitudinal analyses of cognition rarely account for death and dropout, measurement error of the cognitive phenotype, and differing trajectories for different population sub-groups. This paper applies a joint latent class mixed model (and a multi-state model in a sensitivity analysis) that accounts for these issues to a large (n = 3,653), population-based cohort, Paquid, to model the relationship between cognitive lifestyle and cognitive decline. Cognition was assessed over a 20-year period using the Mini-Mental State Examination. Three cognitive lifestyle variables were assessed: education, mid-life occupation, and late-life social engagement. The analysis identified four latent sub-populations with class-specific longitudinal cognitive decline and mortality risk. Irrespective of the cognitive trajectory, increased social engagement was associated with a decreased mortality risk. High education was associated with the most favourable cognitive trajectory, and after adjusting for cognitive decline, with an increased mortality risk. Mid-life occupational complexity was also associated with more favourable trajectories but not with mortality risk. To realistically examine the link between cognitive lifestyle and cognitive decline, complex statistical models are required. This paper applies and compares in a sensitivity analysis two such models, and shows education to be linked to a compression of cognitive morbidity irrespective of cognitive trajectory. Furthermore, a potentially modifiable variable, late-life social engagement is associated with a decreased mortality risk in all of the population sub-groups.

**Electronic supplementary material:**

The online version of this article (doi:10.1007/s10654-014-9881-8) contains supplementary material, which is available to authorized users.

## Introduction

There is increasing evidence to relate measures of cognitive lifestyle, such as education, occupation, and social engagement to late-life cognitive ability and incident dementia in later life [1–4]. Understanding the relationship between these potentially modifiable variables and longitudinal population-based cognitive trajectories is vital as even a modest delay to the onset of severe cognitive impairment and dementia could have a tremendous public health impact [5]. Studies have shown that cognitive lifestyle variables in tandem, but not individually, are linked to a decrease risk of incident dementia [6] via multiple possible pathways [7]. Basic measures of education, occupation, and social engagement have also been linked to a compression of cognitive morbidity in longitudinal analyses [8].

However, there are still some mixed findings, particularly for models that examine education and cognitive change [8–12], which may stem from methodological issues. For example, it is rare for longitudinal analyses of cognitive decline in the older population to account for death and dropout, measurement error of the cognitive phenotype, ceiling and floor effects in the cognitive test [13], and the possibility of cognitive recovery, especially from the mild cognitive impairment state [14]. Furthermore, modelling a single trajectory for a large population will not account for heterogeneous sub-groups with different rates of both initial ability and decline [15]. There may also be differing effects of covariates on these different sub-populations. Other factors to consider include cohort effects when a population age-range spans several years [16], and practice effects when the same cognitive tests are administered over multiple waves [17].

The application of models that account for these issues will enable a more realistic assessment of the disease process and better identify the true effects of cognitive lifestyle on cognitive decline. Two statistical approaches that can help do this are joint latent class models [15] and multi-state models [18]. Previously, a longitudinal multi-state analysis of 13,004 subjects from the Cognitive Function and Ageing Study (CFAS) found cognitive lifestyle factors to be linked to a compression of cognitive morbidity [18]. However, this analysis did not account for population heterogeneity or missing cognitive data. It also required the definition of cognitive states, which both reduces power to detect subtle cognitive changes and limits its application to outcomes that can be translated into clinically meaningful states. In contrast, a joint latent class mixed model uses all of the information from the quantitative cognitive score(s) and can model trajectories and co-occurring events, such as death, in multiple sub-populations. However, it is unable to explicitly model cognitive recovery.

The aim of this paper was to assess (1) the relationship between cognitive lifestyle and late-life cognitive decline accounting for death, and (2) the relationship between cognitive lifestyle and death adjusting for cognitive trajectories in the French cohort, Paquid using a joint latent class mixed model. As a sensitivity analysis and for methodological comparison, a multi-state model was also examined.

## Materials and methods

### Study population

Data stem from the Paquid cohort, which is a longitudinal study of ageing with up to 20 years of follow-up [19]. Recruitment of 3,777 participants occurred across 75 civil parishes of the Gironde and Dordogne regions of south west France. Subjects were aged 65 years and above, residing at home at the study baseline in 1988. There have been up to nine subsequent waves of data collected on each individual at 1, 3, 5, 8, 10, 13, 15, 17, and 20 years after the baseline assessment. At the first follow-up, only subjects from Gironde were interviewed.

### Cognitive assessment

At each wave of Paquid, a multi-test cognitive battery was administered to the participants by a trained neuropsychologist. For this analysis we only consider scores from one of the tests, the Mini-Mental State Examination (MMSE) [20]. The MMSE is a test of global cognitive function that is brief to administer and has a scoring range of 0–30. The MMSE scores were treated as continuous data for the joint latent class mixed model analysis. For the multi-state model of cognitive change, MMSE categories were specified as follows: no impairment was defined as a score between 27 and 30, slight impairment for scores between 23 and 26, and moderate/severe impairment for scores below 23 [18]. An MMSE score between 23 and 26 has been shown to be as effective at predicting conversion to dementia as other more detailed clinical measures of mild cognitive impairment [21].

### Measurement of cognitive lifestyle

Three baseline measurements of cognitive lifestyle were included in this analysis: early-life education, mid-life occupation, and late-life social engagement. Education was split into three groups: no education or a non-validated primary school degree; a validated primary degree up to a non-validated secondary degree; and a validated secondary degree or higher. Mid-life occupation was dichotomised into intellectual (craftsmen and shopkeepers, policemen, nurses and white collar workers, and professional workers) versus non-intellectual type work (housewives, farm workers, farm managers, domestic service employees, and blue collar workers). Four binary response questions were combined to create a scale for late-life social engagement. They focussed on: membership of a group or association; visits from family and friends; membership of a golden age club; and membership of another club. A three category response was created after summing the scores from all four variables: low social engagement (sum score 0); medium social engagement (sum score 1); and high social engagement (sum score 2, 3, or 4). Although a more detailed list of social engagement questions was available for analysis, those chosen are analogous to the questions used in CFAS where such a measure was linked to cognitive decline [18].

### Statistical analysis

**Fig. 1:**
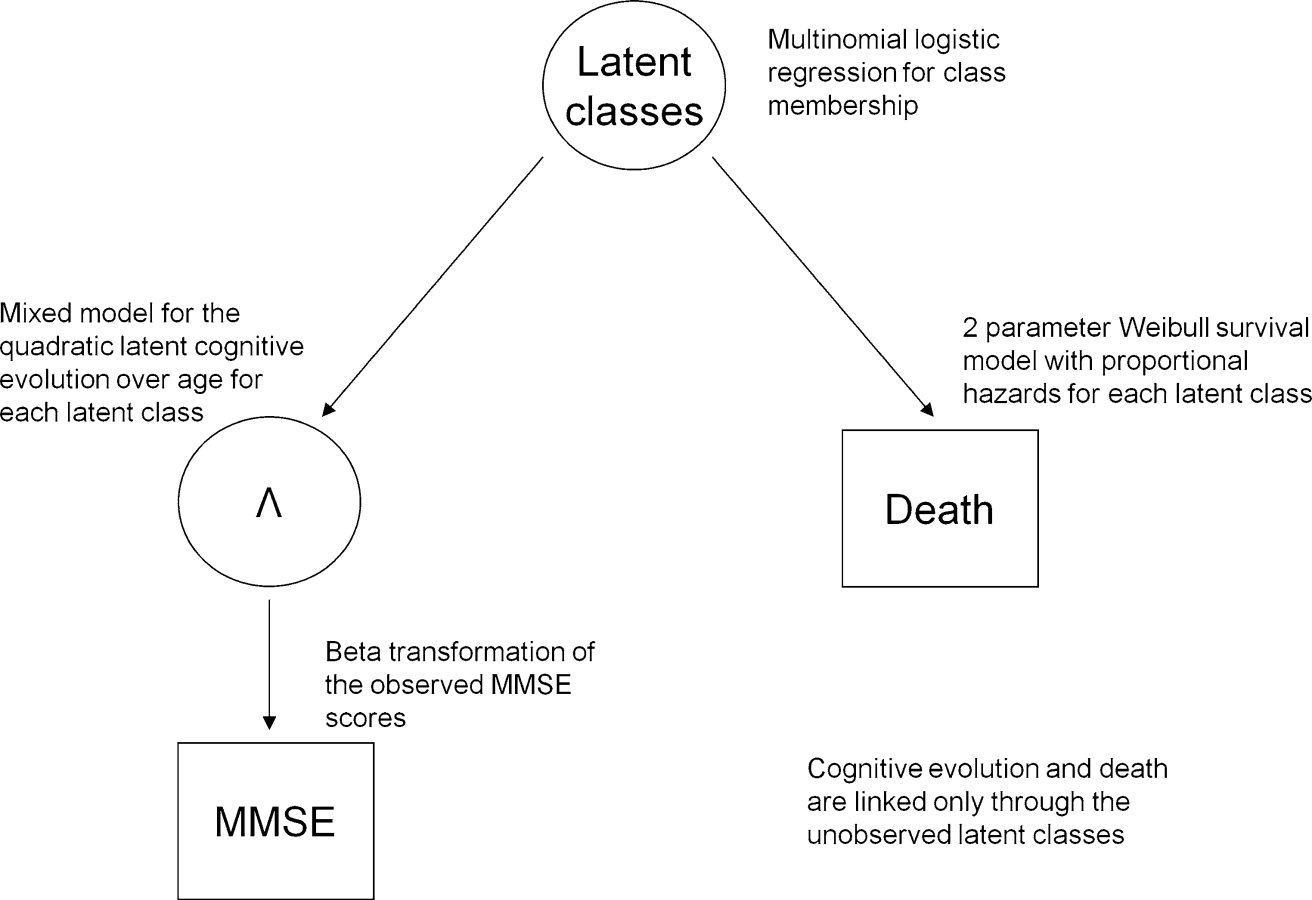
Pictorial representation of the joint latent class mixed model

Data were analysed using a joint latent class mixed model (pictured in Fig. 1). The MMSE was treated as a continuous variable and the changes over time were modelled jointly with death. The model distinguishes sub-populations (latent classes) that are characterised by different profiles of cognitive decline and mortality risk. The probability of latent class membership was explained according to sex, and the three cognitive lifestyle variables. Observed MMSE scores were assumed to be noisy, curvilinear measures of the true latent cognitive level [13]. Curvilinearity refers to the unequal sensitivity of the cognitive test to measure cognitive change. It means that a 1-point change does not represent the same cognitive loss from two different initial levels, and leads to floor/ceiling effects and a non Gaussian distribution of the score [22]. To normalize the observed MMSE scores and to link them to the true latent ability, a parameterized non-linear link function (specifically a Beta cumulative distribution function) [13] was applied. Evolution of the true latent ability was assessed using a latent-class-specific linear mixed model with a class-specific quadratic age trend to account for non-linear cognitive decline; a binary ‘first visit’ variable was included as a common estimate across the classes to account for a potential learning effect between waves one and two but not between waves thereafter [17]. This class common effect assumes that the learning effect is identical for all individuals. Class specific parameters increase the model complexity substantially but allow a more flexible fit. Where these effects were similar e.g., learning effects, these can be treated as class common effects, yielding a more parsimonious model. This can be formally tested for via a multivariate Wald test. Random-effects were included to incorporate individual variation in the intercept and both the linear and quadratic slopes of decline. The joint survival model with death as the time-to-event outcome was defined by a two parameter Weibull distribution with class-specific proportional hazards. Covariates were the same as those used for the class membership (sex and cognitive lifestyle variables) and were assumed to be class specific. However, given that some of the latent class profiles contained too few individuals with some characteristics (high education, for example), education and social engagement were re-coded as binary variables (lowest group versus the two highest groups for both variables) in order to estimate these class-specific parameters. After parameter estimation, the posterior probabilities to belong to each latent class were computed for each subject and they were then assigned to the class with the highest probability [15]. The number of latent classes was determined using the BIC selection criteria as in Proust-Lima et al. [15], a measure of entropy [23, 24], and by assessing the mean posterior probabilities of belonging to each latent class according to the final classification (minimum threshold of 0.65). Analyses were also repeated using different starting values to minimise the chance of the models converging to a local maximum. Data were analysed using a Fortran90 programme developed by Proust-Lima et al. [15], which can be found online (http://etudes.isped.u-bordeaux2.fr/BIOSTATISTIQUE/HETMIXSURV/US-Biostats-HETMIXSURV.html).

### Sensitivity analysis

**Fig. 2:**
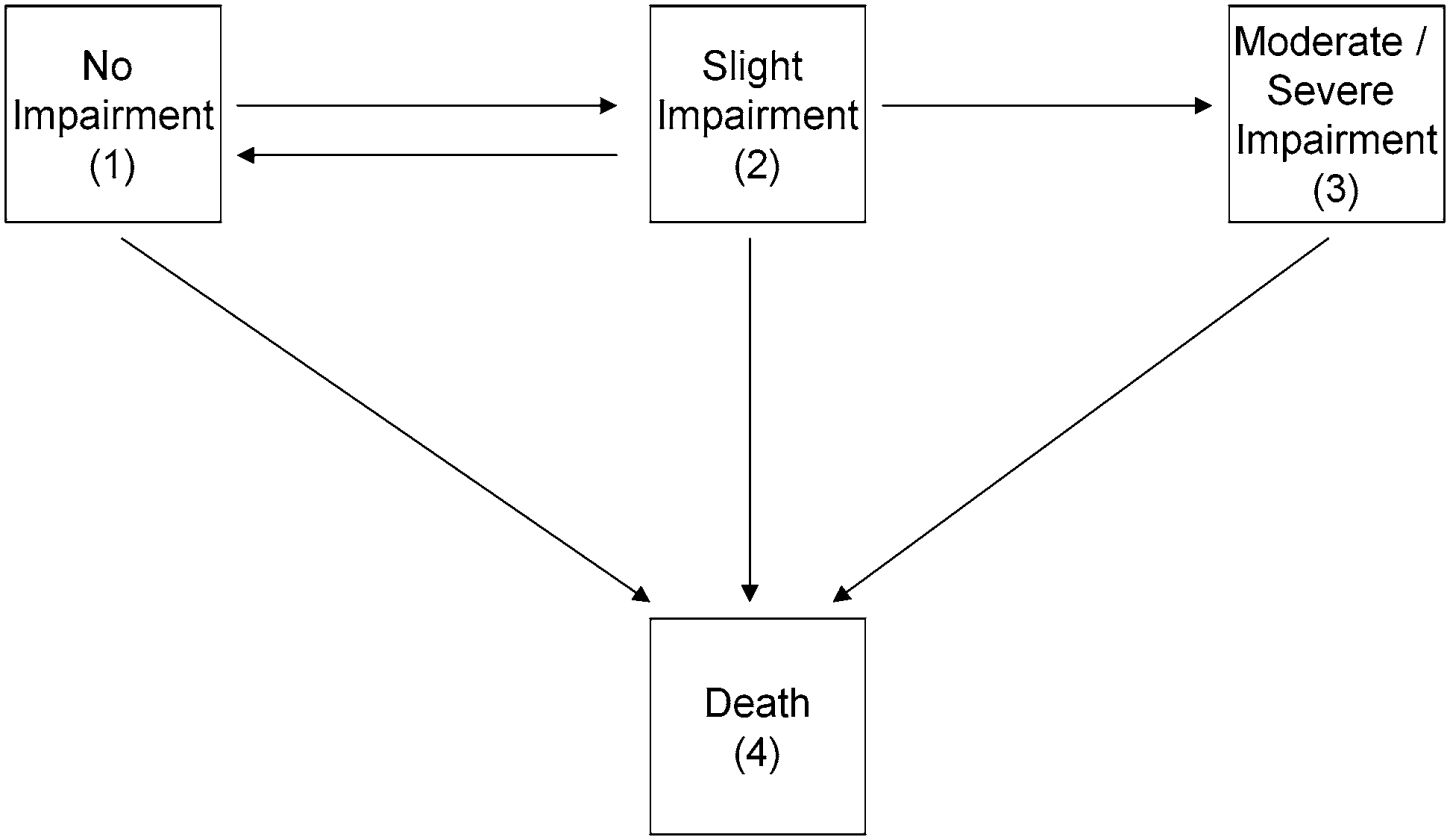
Pictorial representation of the multi-state model. There are six transition specific hazards, q_rs_(t), where r and s are contained within the state set (*1* no impairment, *2* slight impairment, *3* moderate/severe impairment, *4* death) and t represents time. The three cognitive lifestyle covariates plus sex and the time-dependent variable age are linked to each hazard via log-linear regression

As a sensitivity analysis, an analogous multi-state model was built. Full details of the model and its assumptions have been previously published [8, 18]. Briefly, the model (pictured and described in detail in Fig. 2) contained three living states (no cognitive impairment; slight cognitive impairment; moderate/severe cognitive impairment) and death as an absorbing state. Transitions were modelled between adjacent states except for recovery from moderate/severe impairment which was too rare. The exact entry time into each cognitive state is unknown and the transition intensities vary by age, a time-dependent covariate, sex and the three cognitive lifestyle variables. Measurement error of the cognitive states was accounted for by a hidden Markov model that treats the observed MMSE states as potentially misclassified manifestations of the true underlying cognitive state. Back transitions from moderate/severe impairment were assumed to be misclassifications. Further sensitivity analyses were conducted to test for possible cohort effects by adding baseline age as a transition specific covariate and to lag the effect of social engagement by only considering cognitive data from the 5 year follow-up onwards. Data were analysed using R version 2.13.1 [25] and the R package ‘msm’ [26].

## Results

The Paquid cohort is described in Table 1. Excluding 124 participants with missing cognitive lifestyle data, no cognitive follow-up and death/censoring information, or no baseline MMSE score left an analysis sample of 3,653. A total of 458 participants were followed from baseline through to the tenth interview wave and 2,921 (80 %) deaths were observed throughout the duration of the study, leaving 274 people who were lost to follow-up (censored). Only a small proportion of the cohort had a high level of education (validated secondary degree or higher (n = 378, 10.3 %)) with most having no education or a non-validated primary school degree (n = 1,283, 35.1 %) or a validated primary degree up to a non-validated secondary degree (n = 1,992, 54.5 %). There was an even split of mid-life occupational complexity with n = 1,764 (48.3 %) participants classified as having an intellectually demanding job. In terms of late-life social engagement levels, the majority of the group was engaged in moderate activity (n = 1,964, 53.8 %), with n = 1,121 (30.7 %) being highly active and n = 568 (15.5 %) being inactive. A cross-tabulation of the variables by MMSE score is shown in Online Resource 1. Older subjects were more likely to have a lower MMSE score, as were those with lowest levels of education, occupation, and social engagement.

**Table 1:** Description of the Paquid cohort

	Total (n = 3,653)	
Age (mean, s.d.)	75.3	6.8
Sex (n, %)		
Male	1,539	42.1
Education (n, %)		
Low	1,283	35.1
Medium	1,992	54.5
High	378	10.3
Mid-life occupation (n, %)		
Non-intellectual	1,889	51.7
Intellectual	1,764	48.3
Late-life social engagement (n, %)		
Low	568	15.5
Medium	1,964	53.8
High	1,121	30.7
MMSE group (n, %)		
No impairment (27–30)	1,908	52.2
Slight impairment (23–26)	1,039	28.4
Moderate/Severe impairment (0–22)	706	19.3

**Fig. 3:**
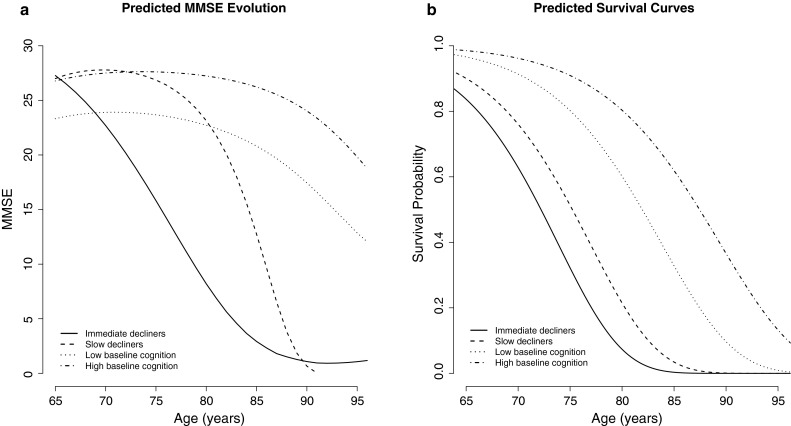
**a** Predicted MMSE evolution over time (age in years) for the four latent classes, **b** predicted survival curves by latent class (for a man with low education, a non-intellectual occupation, and low social engagement)

The joint latent class mixed model with four latent classes had the best fit (Online Resource 2). Model building started with a single class and additional classes were added until the BIC measure of fit was minimised. Models with more than one class were also required to have relatively high mean posterior class membership probabilities (>0.65). The four mean longitudinal cognitive trajectories are illustrated in Fig. 3a. There are two roughly parallel cognitive trajectories, classes 3 (*low baseline cognition,* n = 1,237) and 4 (*high baseline cognition,* n = 1,871), with the latter having a higher initial MMSE score. The two other classes start at the same high cognitive level as class 4 (MMSE ~27) with class 2 (*slow decliners,* n = 412) remaining at this level until around age 75 before declining steeply, while class 3 (*immediate decliners,* n = 133) has a steep, almost linear decline until age 85 by which point nearly all subjects are dead. The probabilities for class membership (the mean probability of being assigned to class *x* for individuals placed in class *x*) were high, ranging from 0.69 to 0.87. However, there was notable uncertainty for the *slow decliners*, where the mean probability of belonging to the *high baseline cognition* group was 0.21. The class-common effect for the first visit was statistically significant in the longitudinal model, indicating worse cognitive scores at baseline visit compared to subsequent visits (β −0.39 S.E. 0.07). This implies that the baseline scores were an average of 0.39 SDs below the mean latent cognitive score.

The covariate profiles of the classes are expressed in Table 2 as the odds of belonging to each class relative to the *high baseline cognition* class for each covariate category relative to the reference category. For example, the odds of belonging to the *immediate decliners* compared to the *high baseline cognition* group was 10 times lower for those with medium education relative to those with low education after adjusting for sex, occupation, and social engagement—odds ratio (OR) 0.1 (95 % CI 0.0, 0.1). For gender, the odds for women to belong to the *slow or immediate decliners* were two times lower than to belong to the *high baseline cognition* group, which had a similar distribution to the *low baseline cognition* group. The *high baseline cognition* group was the most educated with the *immediate decliners* group the least—no individuals with high education were assigned to this class. The distribution of occupational complexity was similar in the *high baseline cognition* and *slow decliners* groups; those in the *low baseline cognition* and *immediate decliners* groups were more likely to have a non-intellectual job. Finally, individuals assigned to all classes apart from the *high baseline cognition* group tended to have lower levels of late-life social engagement. The odds to be placed in the *high baseline cognition* group compared to the *immediate or slow decliners or low baseline cognition* groups were around 10, 3, and 5 times greater for those with high social engagement.

**Table 2:** Joint latent class mixed model output for the association between cognitive lifestyle and cognitive decline and mortality

	Latent class							
	Immediate decliners (1)		Slow decliners (2)		Low baseline cognition (3)		High baseline cognition (4)	
Number assigned to each class	133		413		1,236		1,871	
Class membership probability—OR (95 % CI)								
Sex (female vs. male)	**0.5**	**0.3, 0.8**	**0.6**	**0.5, 0.9**	1.1	0.8, 1.6	Ref	
Education (medium vs. low)	**0.1**	**0.0, 0.1**	**0.4**	**0.2, 0.8**	**0.03**	**0.0, 0.1**	–	
Education (high vs. low)	**0.1**	**0.0, 0.2**	**0.3**	**0.2, 0.7**	0.0	0, ∞	–	
Occupation (intellectual vs. non-intellectual)	**0.3**	**0.2, 0.6**	1.0	1.0, 1.0	**0.2**	**0.1, 0.3**	–	
Social engagement (medium vs. low)	**0.1**	**0.1, 0.3**	**0.3**	**0.2, 0.6**	**0.3**	**0.2, 0.4**	–	
Social engagement (high vs. low)	**0.1**	**0.0, 0.1**	**0.3**	**0.2, 0.6**	**0.2**	**0.1, 0.3**	–	
Risk of death—HR (95 % CI)								
Class-specific intercept	**11.9**	**6.4, 22.3**	**7.0**	**3.5, 14.0**	**2.3**	**1.6, 3.4**	Ref	
Sex (female vs. male)	**0.3**	**0.2, 0.4**	**0.4**	**0.4, 0.6**	**0.5**	**0.4, 0.6**	**0.6**	**0.5, 0.7**
Education (medium|high vs. low)	1.4	0.9, 2.2	1.0	0.6, 1.8	1.2	1.0, 1.5	**1.4**	**1.1, 1.8**
Occupation (non-intellectual vs. intellectual)	0.8	0.5, 1.2	1.0	0.8, 1.3	0.9	0.7, 1.1	1.0	0.8, 1.1
Social engagement (medium|high vs. low)	1.1	0.7, 1.8	**0.7**	**0.5, 1.0**	**0.8**	**0.6, 0.9**	**0.7**	**0.6, 0.9**

Bold values indicate the estimates where *p* < 0.05

Estimates for the survival sub-model are also shown in Table 2 and illustrated in Fig. 3b. The *high baseline cognition* group had the highest survival probabilities whilst the *immediate and slow decliners* had the greatest mortality risk: 11.9 (95 % CI 6.4, 22.3) and 7.0 (95 % CI 3.5, 14.0) times greater than the *high baseline cognition* group. The class-specific hazard ratios (HRs) for survival showed an increased mortality risk for those with a higher level of education, although this was only statistically significant in the *high baseline cognition* group: HR 1.4 (95 % CI 1.1, 1.8). After adjustment, there was no association between occupation and survival but being socially engaged was associated with a decreased mortality risk in all except the *immediate decliners* (HR range 0.7–0.8). Simplification of the model to allow class-common effects of education and social engagement on survival resulted in an increased mortality risk for those with medium and high education—HRs 1.3 (95 % CI 1.1, 1.4) and 1.2 (95 % CI 1.0, 1.4), respectively—and a decreased mortality risk for those with medium or high social engagement—HRs 0.8 (95 % CI 0.7, 0.9) and 0.7 (95 % CI 0.6, 0.8), respectively.

### Sensitivity analyses

These findings were compared to those obtained by a multi-state model. Results are presented in Table 3. Compared to those with low education, subjects with high education had half the risk of moving to a slightly impaired state—HR 0.5 (95 % CI 0.3, 0.7); around a 30 times greater chance of cognitive recovery from slight impairment back to no impairment—HR 27.3 (95 % CI 9.6, 77.5); and one-and-a-half times the mortality risk from moderate/severe impairment—HR 1.5 (95 % CI 1.1, 2.1). Similar but attenuated associations were found for the medium educated group. The effects of occupation on the transitions were in the same direction as those for education but of a much smaller magnitude. There was also a small increase in the risk of moving from slight impairment to moderate/severe impairment for those with an intellectual (versus non-intellectual) occupation. Finally, medium and high levels of late-life social engagement were associated with a decreased mortality risk from all three cognitive states—HRs for high versus low social engagement were 0.6 (95 % CI 0.4, 0.8) for those with no impairment; 0.5 (95 % CI 0.3, 0.7) for those with slight impairment; and 0.8 (95 % CI 0.6, 0.9) for those with moderate/severe impairment.

**Table 3:** Hazard Ratios and 95 % CIs for education, mid-life occupation and late-life social engagement on late-life cognitive change

Covariate transition	Education (med vs. low)		Education (high vs. low)		Occupation (int vs. non-int)		Social engagement (med vs. low)		Social engagement (high vs. low)	
State 1—State 2	**0.7**	**0.6, 0.9**	**0.5**	**0.3, 0.7**	**0.8**	**0.6, 1.0**	0.9	0.6, 1.2	0.8	0.6, 1.1
State 1—Death	0.9	0.6, 1.2	0.9	0.6, 1.2	1.0	0.8, 1.2	**0.6**	**0.5, 0.9**	**0.6**	0.4, 0.8
State 2—State 1	**10.0**	**4.6, 21.7**	**27.3**	**9.6, 77.5**	**3.1**	**1.8, 5.1**	1.0	0.4, 2.6	2.1	0.8, 5.3
State 2—State 3	1.0	0.8, 1.2	1.1	0.7, 1.7	**1.3**	**1.0, 1.5**	1.1	0.8, 1.5	1.0	0.8, 1.4
State 2—Death	1.0	0.8, 1.4	1.0	0.5, 2.1	0.9	0.7, 1.2	**0.5**	**0.4, 0.8**	**0.5**	0.3, 0.7
State 3—Death	**1.5**	**1.3, 1.7**	**1.5**	**1.1, 2.1**	1.1	0.9, 1.2	**0.8**	**0.7, 1.0**	**0.8**	0.6, 0.9

State 1 (no impairment): MMSE 27-30, State 2 (slight impairment): MMSE 23-26, State 3 (moderate to severe impairment): MMSE 0-22

Bold values indicate the estimates where *p* < 0.05

Two additional sensitivity analyses examined potential cohort effects and lagged effects of social engagement. The former included baseline age as a covariate on each state transition. This had no effect on the associations for the cognitive lifestyle covariates (results not shown). Similarly, a model that considered cognitive decline and mortality 5 years after the baseline assessment found similar protective effects of social engagement on transitions to death (results not shown).

## Discussion

This study applied two statistical models to a large, population-based cohort with up to 20 years of follow-up. They found late-life social engagement to be strongly associated with a decreased mortality risk irrespective of cognitive decline profile or cognitive state (no, slight, or moderate/severe impairment). By contrast, high education was associated with an increased mortality risk irrespective of cognitive trajectory. It was also associated with the most favourable cognitive trajectory in the mixed model, and with a reduced risk of cognitive decline in the multi-state model. In both models, mid-life occupation tended to be associated with more favourable cognitive change but not with mortality.

These findings are compatible with a cognitive reserve hypothesis [27]. We found a compression of cognitive morbidity in participants who had high levels of education, a non-manual occupation in mid-life, and an active social engagement status in late-life. This could be due to neurocompensation i.e., the provision of coping strategies that alleviate the impact of underlying damage until this becomes overwhelming, resulting in an accelerated terminal decline.

The multi-state modelling results for education and occupation closely resemble those reported previously in a UK-based cohort [18]. However, there was no overlap in the findings for social engagement. Possible reasons for this include sampling differences: at baseline CFAS included subjects living in both the community and in institutions and had an 81 % response rate, Paquid included only community-dwelling subjects and had a 68 % response rate. The use of a single measure of social engagement at baseline is a limitation in both studies.

### Comparison of the statistical models

While the interpretation of the results for the multi-state model and the joint latent class mixed model are similar, relative strengths and weaknesses of the approaches can still be discussed. The main benefits of the joint latent class mixed model are three-fold. Firstly is its ability to use all available information from quantitative response variables, which avoids the need to generate cut-points and to define states. Moreover, extension of the latent class model can allow the combination of information from multiple tests to create a general cognitive factor that evolves over time [15]. Secondly the model accounts for population heterogeneity by identifying latent classes and display underlying trajectories of cognitive decline. However, individuals are not allowed by the model to change latent class. Thirdly, the joint latent class model is particularly robust to missing data. It explicitly deals with death, and missingness due to dementia dropout is handled via the latent cognitive trajectories. An advantage of using either method for longitudinal analyses of MMSE scores is that they overcome curvilinearity via cognitive state definitions or a nonlinear transformation of the MMSE scores. Both methods are also able to model death. Despite commonly being used in longitudinal models of cognitive ageing [8, 11, 12], a potential limitation is our use of the MMSE. It is typically used as a screening tool for dementia prediction, which provides rationale for its use in the multi-state model. Furthermore, while it is insensitive to change at the ceiling and floor of the distribution [13], our mixed modelling approach was able to account for this.

Limitations of the joint latent class mixed model analysis include a less intuitive interpretation of the results compared to the multi-state model, which yields a single set of HRs for each covariate. A unique feature of the multi-state model was the explicit modelling of cognitive recovery from slight impairment back to no impairment. An alternative approach to the joint latent class model could be a two-stage approach where a latent class mixed model would be estimated first using only repeated measures of MMSE to identify the subpopulations and then a survival model would be estimated in each posterior class. However, the joint model is a better option whenever possible because it accounts for truncation by death when estimating the trajectories and because variances of the regression parameters in the survival model are better estimated by taking into account the uncertainty of the estimates of the latent class mixed models.

Both multi-state models and joint latent class mixed models have flexible properties that enable a realistic modelling of longitudinal cognitive decline although subtle differences exist in terms of interpreting output. For future analyses the most appropriate model will depend on the hypothesis to be tested, the cognitive test under study and the study design. If the objective is to study cognitive recovery or life expectancies, and if clinically meaningful cognitive states can be defined then the multi-state model is ideal. On the other hand, a joint latent class mixed model, which uses all of the information provided by quantitative scores, is ideal when change in the dependent variable is likely to be small, when cognitive states are difficult to define, or when multiple cognitive outcomes are being assessed simultaneously.

## Conclusion

There are two main epidemiological findings from this analysis. Firstly, social engagement was associated with a decreased mortality risk in Paquid. While causality cannot be implied from this analysis, a model that considered cognitive decline and mortality 5 years after the assessment of social engagement found similar effects. Hence, even if it is only a marker of general health and well-being, social engagement may be a useful predictor of mortality risk. Secondly, there is evidence to link education to a decreased risk of cognitive decline but an increased mortality risk from severe cognitive impairment. These findings were also observed in CFAS [18], with the latter finding also replicated in the US Health and Retirement Study [28]. The former finding is in contrast with some but not all previous analyses where education has been linked to initial cognitive level but not the rate of decline [12, 29, 30]. However, there are major methodological problems related to the analysis of cognitive decline of the older population, such as competing risks for mortality, missing data, unequal sensitivity to change (with floor and ceiling effects), cognitive recovery, and measurement error. The application of methods that account for these issues is vital in order to provide a more valid and accurate assessment of cognitive ageing.

## Electronic supplementary material

Below is the link to the electronic supplementary material.

Supplementary material 1 (DOCX 16 kb)
