# Supplementary material for: Cognitive lifestyle jointly predicts longitudinal cognitive decline and mortality risk
Source: Eur J Epidemiol. 2014 Feb 28;29(3):211–9. doi: 10.1007/s10654-014-9881-8 (PMC4003346; doi:10.1007/s10654-014-9881-8)
Supplement: Supplementary file 1 — Supplementary material 1 (DOCX 16 kb) [file 10654_2014_9881_MOESM1_ESM.docx]

Online Resource 1. Summary of Cognitive State Transitions

| To  From | 1 | | 2 | | 3 | | Death | | Censored | |
| --- | --- | --- | --- | --- | --- | --- | --- | --- | --- | --- |
| 1 | 5,870 | (69%) | 1,252 | (15%) | 218 | (3%) | 1,066 | (12%) | 137 | (2%) |
| 2 | 890 | (22%) | 1,480 | (37%) | 674 | (17%) | 857 | (22%) | 77 | (2%) |
| 3 | 69 | (3%) | 341 | (13%) | 1,154 | (44%) | 998 | (38%) | 60 | (2%) |

State 1 (no impairment): MMSE 27-30, State 2 (slight impairment): MMSE 23-26, State 3 (moderate to severe impairment): MMSE 0-22

Online Resource 2: Summary of Model Fit

| N classes | BIC | Posterior classification probabilities | Class Size | Entropy |
| --- | --- | --- | --- | --- |
| 3 | 87,511.60 | 0.71, 0.85, 0.88 | 348, 1279, 2026 | 1.31 |
| 4 | 87,299.10 | 0.78, 0.69, 0.84, 0.87 | 133, 413, 1236, 1871 | 1.29 |
| 5 | 87,227.50 | 0.83, 0.56, 0.80, 0.84, 0.71 | 1247, 199, 131, 1808, 268 | 1.29 |
